# Gallium-68 Prostate-Specific Membrane Antigen Positron Emission Tomography: A Practical Guide for Radiologists and Clinicians

**DOI:** 10.7759/cureus.22917

**Published:** 2022-03-07

**Authors:** Fathima Fijula Palot Manzil, Harleen Kaur, Lajos Szabados

**Affiliations:** 1 Radiology/Nuclear Medicine, University of Arkansas for Medical Sciences, Little Rock, USA; 2 Radiology/Nuclear Medicine, Hamad General Hospital, Doha, QAT

**Keywords:** prostate-specific membrane antigen, nuclear medicine imaging, molecular imaging for prostate cancer, gallium-68 pet/ct, psma pet

## Abstract

Prostate cancer (PCa) is the third most common form of cancer and the most common cancer diagnosis in men in the United States. It is known that prostate-specific membrane antigen (PSMA) is overexpressed in PCa. PSMA is a type II transmembrane glycoprotein expressed in several benign and malignant tissues. In December 2020, FDA approved Gallium-68 (68Ga) PSMA, which is a PSMA-targeted positron emission tomography (PET) imaging agent. Molecular imaging targeting PSMA has shown substantial advancement in PCa imaging. In this article, we discuss the radiopharmaceutical, indications to do PSMA PET, technical aspects of PSMA PET imaging, normal biodistribution of PSMA, other benign and malignant conditions that can take up PSMA, staging of prostate cancer, and how to report PSMA PET.

## Introduction and background

Prostate-specific membrane antigen (PSMA) is a glutamate carboxypeptidase II metalloproteinase primarily present in prostatic tissues [1]. Increased PSMA expression is seen in a variety of malignancies, most notably in prostate cancer (PCa) [2]. The majority of primary and metastatic prostate adenocarcinomas demonstrate PSMA expression. PSMA expression in PCa increases as cancer aggressiveness increases as marked by higher Gleason scores and morbidity rates [3]. Immunohistochemical studies show that PSMA expression upsurge in hormone-refractory, dedifferentiated, or metastatic PCa and may negatively correlate with survival outcomes. PSMA positron emission tomography (PET)/computed tomography (CT) is used to guide management decisions in clinical practice because of its utility in prognostics [4,5].

PCa recurrence is usually manifested as an increase in prostate-specific antigen (PSA), termed as biochemical recurrence (BCR). BCR often occurs months to years before there is recurrent or residual disease evidence on conventional imaging, limiting treatment decisions. PSMA PET demonstrates much higher sensitivity in detecting residual, recurrent, or metastatic disease, thus leading to key changes in the management of patients with BCR. Conventional imaging for staging PCa includes computed tomography (CT), magnetic resonance imaging (MRI), and 99mTc-labeled phosphate bone scintigraphy, all of which have low sensitivity compared to PSMA PET, particularly at low PSA levels [6]. PSMA PET/CT may lead to management changes in a high proportion of patients after unrevealing conventions [7].

PSMA affect several key oncogenic pathways in the pathogenesis of PCa, specifically the PI3K/Akt pathway [8,9]. Thus, PSMA can drive crucial oncogenic pathways such as cell survival, proliferation, cell migration, and angiogenesis, which signifies the clinical applicability of PSMA as a therapeutic target.

## Review

Radiopharmaceutical

Gallium-68 (68Ga) PSMA-11 is the mainstay radiopharmaceutical described in this article. Fluorine-18 DCFPyL is another PSMA-targeted imaging agent that is currently approved. The diagnostic characteristics of both these PSMA targeting PET agents are comparable as per current knowledge, although there may be small differences.

68Ga is obtained from a 68Ge (Germanium)/68Ga generator system. 68Ga has a half-life of 67.63 minutes and decays by positron emission with an 89% yield. 68Ga PSMA should be manufactured under good manufacturing practice conditions. Quality control should follow the applicable governing pharmacopoeia monograph or national regulations. The suggested dosage is 1.8-2.2 MBq (0.049-0.060 milliCurie) per kilogram bodyweight [10]. The dose is administered intravenously. Variable elution efficiencies during the lifetime of a 68Ge/68Ga generator and short half-life of 68Ga can cause a disparity in injected activities. Following the injection of a radiotracer, subsequent flushing should be done with at least the same volume of 0.9% saline to maximize the dispensed activity.

Indications for PSMA PET

Increasing PSA Levels

Increasing PSA levels is called biochemical recurrence in patients who are already treated for primary cancer with either radical prostatectomy or radical radiotherapy. PSMA PET in this case is recommended especially if PSA values are low between 0.2 and 10 ng/mL to identify potential recurrence or metastases. After radical prostatectomy, BCR is defined as a PSA increase of more than or equal to 0.2 ng/mL measured at 6-13 weeks post-surgery and confirmed by a repeat PSA level of >0.2 ng/mL [11]. After definitive radiation therapy, BCR is defined as a PSA rise of more than or equal to 2 above the nadir attained after radiotherapy, regardless of androgen deprivation therapy (ADT) [12]. PSMA PET shows high sensitivity in these cases if the PSA doubling times are shorter and if the initial Gleason scores were high. Thus, PSMA PET/CT aids to guide salvage therapies such as pelvic lymph node dissection or radiotherapy to the metastatic lesions [13].

Staging Patients With High-Risk Primary PCa

Another indication is to stage patients with high-risk primary prostate cancer (Gleason score > 7, PSA > 20 ng/mL, and clinical stage T2c-3a) before definitive therapy such as surgery or external beam radiation [14]. The chance for lymph node and bone metastases is more in these patients. As compared to conventional imaging such as CT, MRI, or bone scan, 68Ga PSMA PET/CT has a superior role for detecting metastases and thus for initial staging. Detecting occult metastases not spotted on CT or MR affects patient management considerably. A contrast-enhanced 68Ga PSMA PET/CT can replace abdominopelvic CT for the detection of lymph node metastases [10]. However, a pelvic MRI cannot be substituted for detailed local tumor delineation [15]. PSMA PET/CT is more precise for the detection of bone metastases as per preliminary data. Further studies need to be done to know whether additional functional imaging such as Tc-99m MDP bone scan or 18FNaF PET/CT to detect bone metastases has relevant added value after the performance of PSMA PET/CT, especially in patients with a negative PSMA PET scan.

To Target Biopsy

PSMA PET can be used to target biopsy in patients who had prior negative biopsies but with increasing PSA levels and high suspicion of PCa [16,17]. Diagnostic confidence is potentially increased by correlation with local anatomic imaging such as diagnostic MRI.

For Follow-up During and After Systemic Treatment

**Figure 1:**
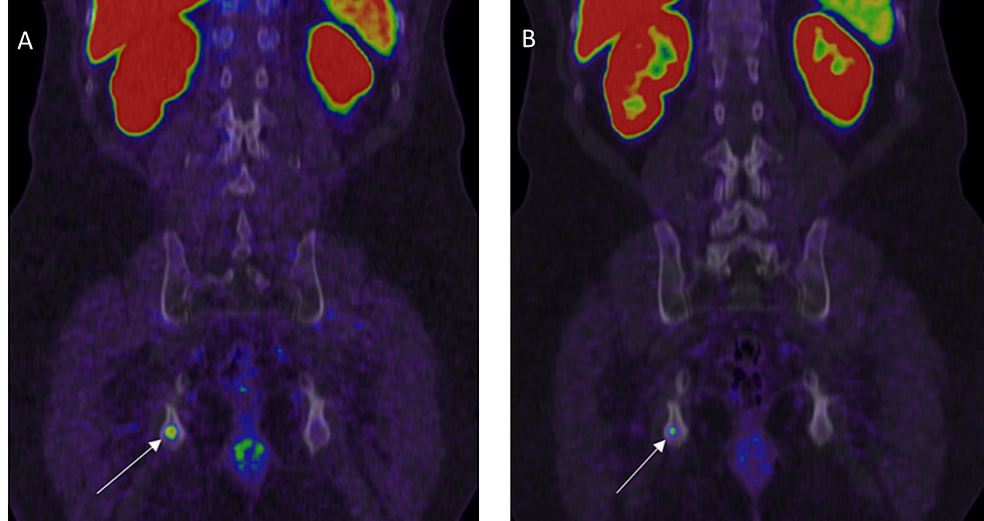
Therapy response A: 68Ga PSMA PET/CT coronal image shows single bone metastasis in the right ischium (white arrow). The lesion was irradiated in this patient on systemic chemotherapy. B: PET/CT six months later shows decreasing uptake of irradiated right ischium metastasis with residual activity denoting viable tumor (white arrow).

PSMA PET helps in the follow-up of patients with metastatic PCa during and after systemic treatment [18] (Figure 1A, 1B). CT scan or MRI could be limited by small metastatic lymph nodes that do not fall under the anatomic imaging size criteria for metastasis. Doubtful sclerotic bone lesions on bone scintigraphy, CT, or MRI could be evaluated on PSMA PET looking for radiotracer uptake in the lesions. Also, bone scintigraphy could be limited by the posttreatment “flare phenomenon.” There are specific effects of each therapy, especially ADT and immunotherapy, on PSMA expression, leading to potential imprecisions and pitfalls, and hence, PSMA PET in these cases should be analyzed with caution according to the time point after the initiation of therapy [19,20].

Staging Before and Monitoring Response During Radioligand Therapy (RLT)

PSMA PET can be used for staging before and monitoring response during 177 Lutetium-PSMA-617 RLT mainly in patients with metastatic castration-resistant PCa [21]. PSMA PET/CT imaging before RLT is important to determine the presence and intensity of PSMA expression in potential target lesions. Based on promising yet preliminary evidence, RLT is offered worldwide at multiple centers within clinical trials or under local regulations. Promising trials are underway to support the immediate future implementation of RLT for the management of PCa. Low PSMA expression in the lesions on the PET scan poses a contraindication for RLT.

Contraindications for PSMA PET

There are no absolute contraindications. Claustrophobia or patient refusing the imaging are relative contraindications. Thyroid and renal function should be taken into account if doing a diagnostic CT using intravenous iodinated contrast along with PET.

Patient preparation

Fasting is not required. Patients should be well hydrated and should void just before image acquisition. High residual activity may be seen in the urinary system of some patients, leading to halo artifacts in PET. Halo artifacts are due to the biodistribution of PSMA tracer resulting in a large organ-to-background activity ratio (OBR) for bladders and kidneys with a positive correlation between OBR and halo size and due to inaccurate or overcorrection for scatter [22]. If not compensated for the halo artifact, false-negative findings can result. Also, activity in ureters might lead to false-positive findings. The administration of 20 mg intravenous furosemide shortly before or after the administration of the tracer may be useful to minimize the halo artifact. Furosemide should not be given in case of allergy or contraindications to furosemide.

Patients can take all their medications. However, the clinical influence of ADT on PSMA PET needs to be considered.

Uptake time

An average of a 60-minute interval is recommended between injection and beginning of imaging. A 50- to 100-minute uptake time is acceptable. There are reports of increased lesion detection with delaying imaging up to three to four hours after injection. Nevertheless, more than a 100-minute uptake time may be concerning due to practicality issues and the short half-life of the radiotracer. If there are indeterminate findings on the one-hour scan, a delayed scan may help identify more lesions, especially those lesions nearer to the urinary bladder or ureters or kidneys, or lesions that show slow PSMA radiotracer accumulation due to low PSMA expression. The time interval between radiotracer injection and the start of imaging should be recorded [10].

PET/CT technical considerations

Integrated PET/CT system is a combination of PET and CT allowing the sequential acquisition of the functional PET and the anatomic CT components. The patient should remain in the same position during both examinations. Usually, the field of view covers the skull base to the mid-thigh. Extended imaging including the entire skull cranially and up to the toes caudally can be done if clinically indicated or if disseminated disease is suspected. Attenuation correction and scatter correction are performed using the CT data. Low-dose CT with low voltage and/or current of the X-ray tube settings is performed commonly. Diagnostic CT using higher voltage and current with or without intravenous and/or oral contrast can be done as per clinical indication. Quantification of 68Ga PSMA PET/CT can be done by measuring relative PSMA concentrations using the standardized uptake value (SUV). The SUV is the most commonly used semiquantitative parameter in PET imaging. There are only scarce data dealing with quantification for 68Ga PSMA. The SUV can be normalized to lean body mass, body weight, or body surface area. With different modes of normalization, SUV values can substantially vary. Hence, serial examinations should use the same mode. The suggested tumor uptake metric is the maximum SUV (SUVmax) and can be measured and documented for target lesions. The SUVmax is the SUV of a single voxel with the highest uptake in a specific lesion on the attenuation corrected PET image. SUVmax values are found to correlate significantly with the grade groups of primary PCa [23]. Quality control steps should be regularly performed to preserve high image quality.

Image acquisition

The patient should remove all removable metal parts from the body/clothing before the imaging starts. Grossly, the coverage of the PET scan should be similar to that of the CT scan of the PET/CT. If the patient can tolerate, imaging should be done with the arms elevated to eliminate artifacts from imaging with arms down. If the PET/CT is performed for radiation therapy planning purposes, imaging should be done in a similar position using the same positioning devices as for radiation treatment. Ideally, the CT scan is performed first from the skull base to the mid-thigh and then subsequently PET scan with the patient remaining in the same position. PET acquisition usually starts caudally from the mid-thigh and proceeds cranially to the skull base to exploit the reduced tracer activity in the bladder after pre-scan voiding and to minimize the proposed misalignment of the urinary bladder due to bladder filling up between CT and PET components.

CT parameters should be in accordance with the clinical requirements and institutional protocols. Usually, PET scans are obtained in 3D mode and two to four minutes per bed position acquisition time. The patient should not move during the total approximate 10- to 30-minute acquisition time.

Image reconstruction

Once PET images are acquired, appropriate corrections are performed for attenuation, scatter, and random coincidences. CT scan is used for attenuation correction. Attenuation corrected and non-attenuation corrected reconstruction should be carried out to recognize possible artifacts produced by the correction algorithm. Appropriate labeling of the reconstructed images should be done. Images are then stored in the local picture archiving and communication system (PACS) [10]. For image analysis, the datasets are usually sent to dedicated post-processing workstations.

Biodistribution of PSMA

Knowing the normal biodistribution of 68Ga PSMA is important to avoid false-positive interpretations. PSMA is a folate hydrolase expressed in a variety of normal tissues and other benign and malignant tumor types despite the “prostate-specific” term. 68Ga PSMA is primarily excreted through the urinary system and a small proportion through the hepatobiliary system.

**Figure 2:**
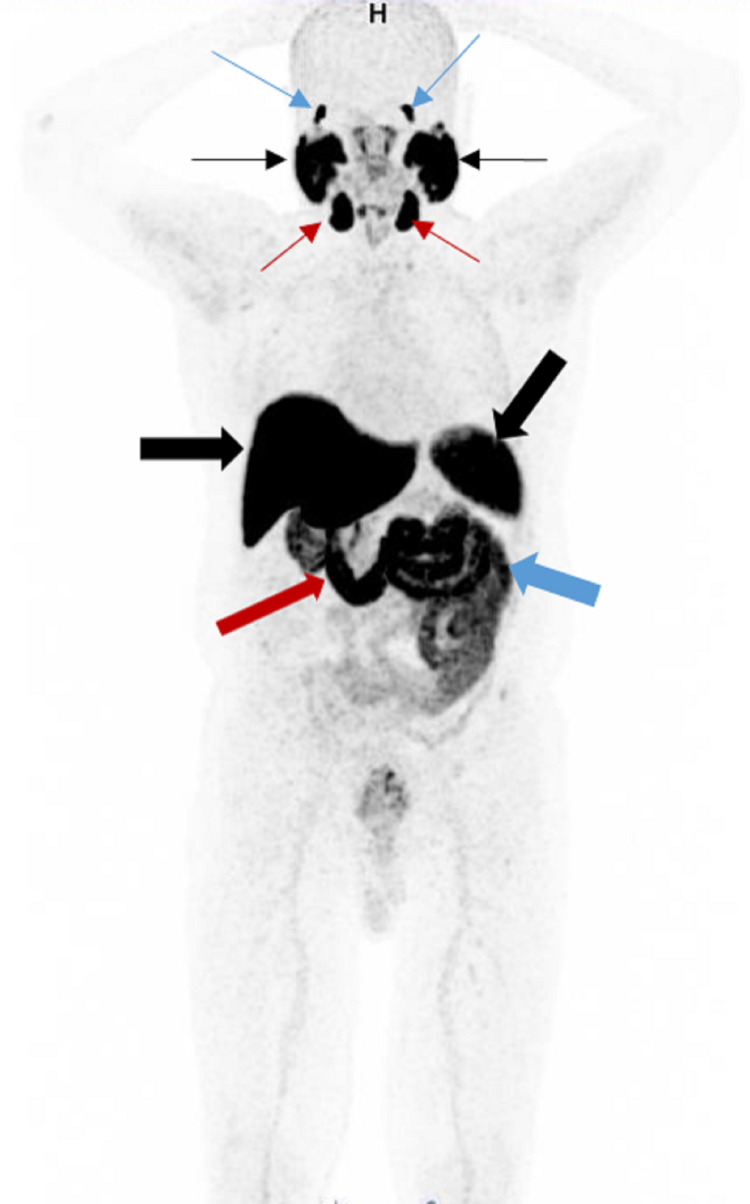
Normal biodistribution Maximum intensity projection (MIP) image shows normal 68Ga PSMA biodistribution with intense radiotracer uptake in the bilateral lacrimal glands (blue arrows), parotid glands (black thin arrows), and submandibular glands (red thin arrows). There is also physiological activity seen in the liver (horizontal black thick arrow), spleen (oblique black thick arrow), duodenum (red thick arrow), and bowel (blue thick arrow).

**Figure 3:**
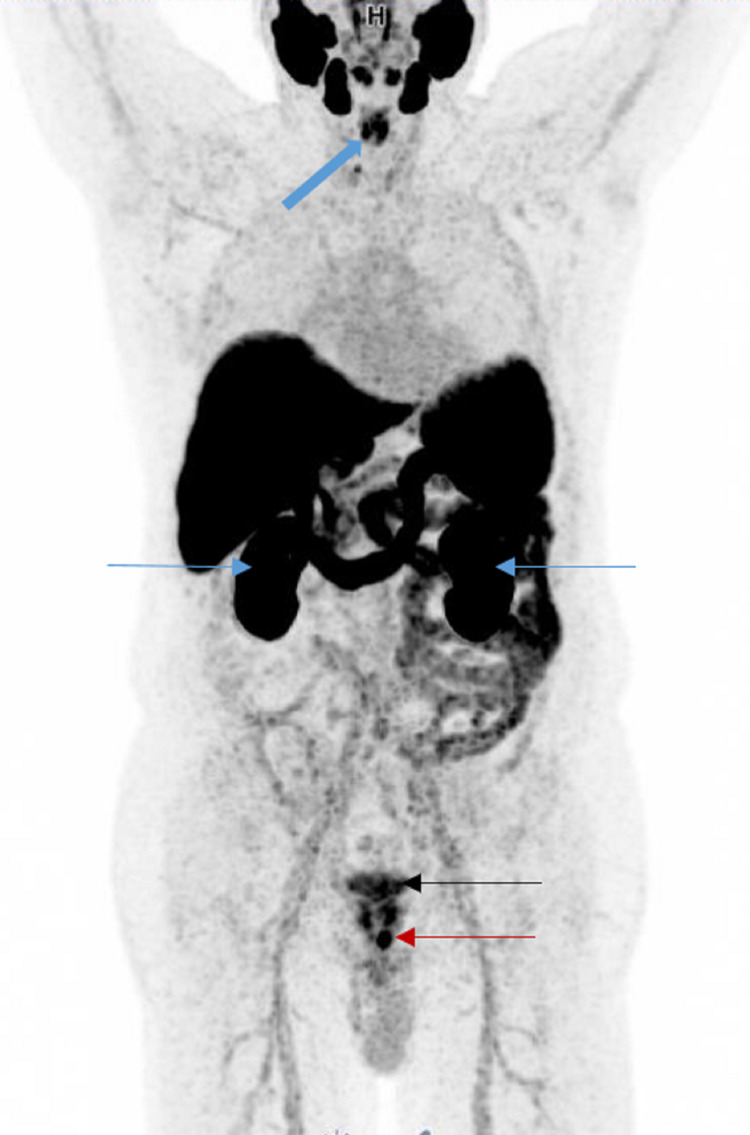
Normal biodistribution 68Ga PSMA MIP image shows physiological activity in the kidneys (blue thin arrows) and urinary bladder (black arrow) from excretion. Note also the physiological activity in the oropharyngeal mucosa (blue thick arrow). Local tumor recurrence is seen in the prostatic bed (red arrow).

**Figure 4:**
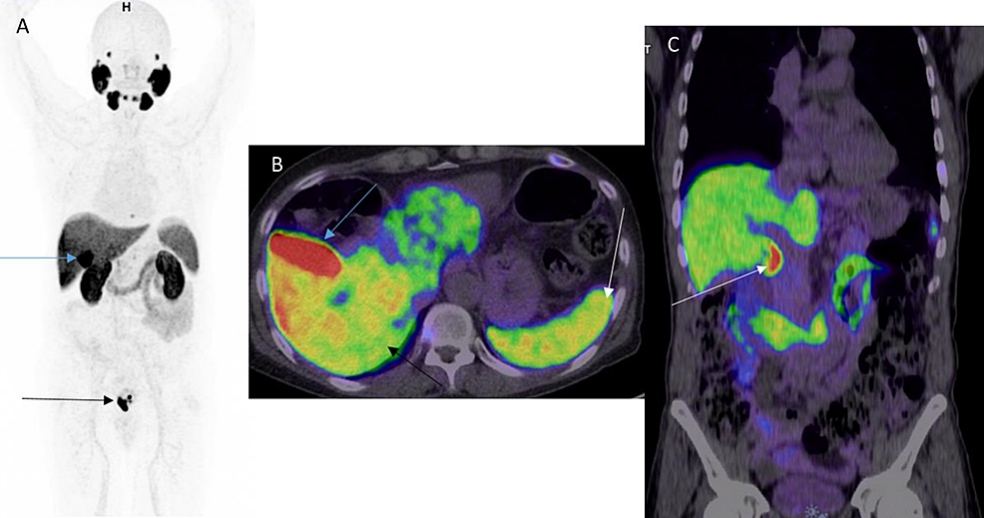
Normal distribution in the gallbladder and bile duct A: 68Ga PSMA MIP image shows physiological activity in the gallbladder (blue arrow). Note the cancer recurrence in the prostatic bed (black arrow). B: Axial image of the upper abdomen shows intense physiological activity in the gallbladder (blue arrow). Moderate uptakes are seen in the liver (black arrow) and spleen (white arrow), which are physiological. C: Coronal image of the chest and abdomen shows intense physiological activity in the common bile duct (white arrow). The uptake correlates to the bile duct on CT (not shown).

**Figure 5:**
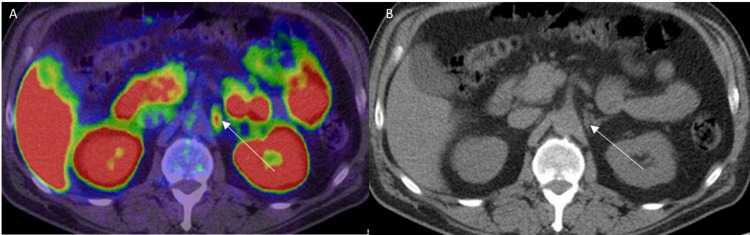
Normal PSMA uptake in the celiac ganglia A: 68Ga PSMA PET/CT axial image of the abdomen shows physiological uptake in the left celiac ganglia (white arrow). B: Correlate CT shows ovoid-shaped left celiac ganglia (white arrow).

**Figure 6:**
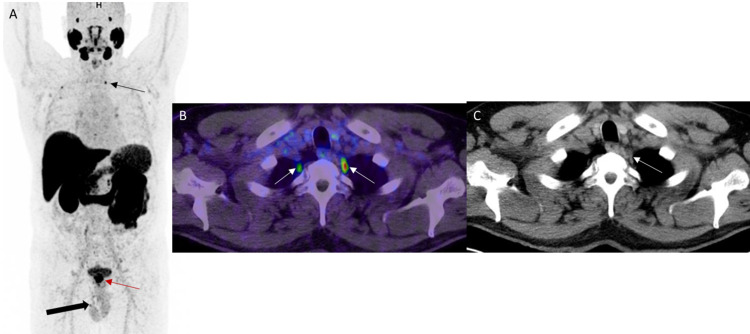
Normal uptake in the stellate ganglia A: MIP image shows uptake in the left stellate ganglion (black thin arrow). Note the pathological uptake in the prostate bed (red arrow). There is mild physiological activity at the testes (black thick arrow). B: PET/CT axial image of the neck and upper chest shows uptake in the bilateral stellate ganglia, left more than right (white arrows). C: CT component of PET/CT shows correlate ovoid-shaped left stellate ganglia (white arrow).

**Figure 7:**
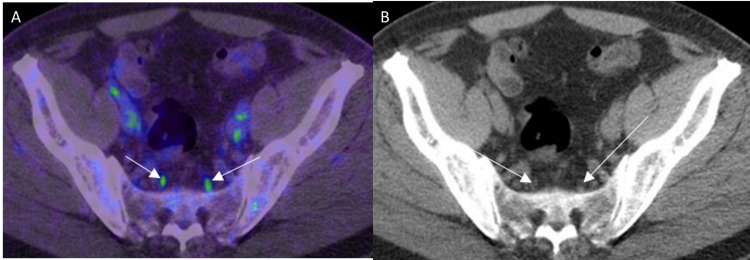
Normal PSMA uptake in the sacral ganglia A: PSMA PET/CT axial image through the pelvis shows radiotracer uptake in the bilateral sacral ganglia (white arrows). B: CT component of PET/CT shows correlate tiny teardrop-shaped soft tissue in the bilateral pre-sacral regions (white arrows) suggestive of the sacral ganglia.

Intense physiological uptake is seen in the lacrimal, parotid, and submandibular glands (Figure 2) [24]. Strong uptake is also seen in the kidneys and urinary bladder due to excretion (Figure 3). Sometimes, activities can be seen in the gallbladder and bile ducts (Figure 4A-4C). Moderate to intense activity will be seen in the liver, spleen, and duodenum (Figure 2) [25]. Variable uptakes can be seen along the oropharyngeal, laryngeal, and esophageal mucosa due to salivary excretion and in celiac (Figure 5A, 5B), stellate (Figure 6A-6C), and presacral (Figure 7A, 7B) ganglia. Variable physiological activity can be seen in the mediastinal blood pool, thyroid, bowel, bone marrow, and testes.

Abnormal PSMA expression in PCa increases progressively in higher-grade tumors, hormone-refractory prostate cancer, and metastatic disease.

Prostate cancer staging

The American Joint Committee on Cancer (AJCC) TNM staging system was updated in 2018 (Table 1). The TNM system for PCa is based on five main categories, which include the extent of the primary tumor (T), regional lymph node involvement (N), metastasis (M), PSA level at the time of diagnosis, and grade (Table 2) based on the Gleason score.

**Table 1:** AJCC staging of prostate cancer

	TNM staging	Grade group	PSA
Stage 1	cT1N0M0	1	<10
	cT2aN0M0	1	<10
	pT2N0M0	1	<10
Stage IIA	cT1N0M0	1	PSA at least 10 but <20
	cT2a or pT2N0M0	1	PSA at least 10 but <20
	cT2b or cT2cN0M0	1	PSA < 20
Stage IIB	T1 or T2N0M0	2	PSA < 20
Stage IIC	T1 or T2N0M0	3 or 4	PSA < 20
Stage IIIA	T1 or T2N0M0	1–4	PSA at least 20
Stage IIIB	T3 or T4N0M0	1–4	Any PSA
Stage IIIC	Any T, N0M0	5	Any PSA
Stage IVA	Any T, N1M0	Any grade group	Any PSA
Stage IVB	Any T, any N, M1	Any grade group	Any PSA

**Table 2:** Grade groups of prostate cancer

Grade	Grade group
Low grade	Grade group 1 (Gleason score ≤ 6)
Intermediate grade	Grade group 2 (Gleason score 3 + 4 = 7)
	Grade group 3 (Gleason score 4 + 3 = 7)
High grade	Grade group 4 (Gleason score 8)
	Grade group 5 (Gleason score 9–10)

Gleason score

The most predominant pattern in the pathology on prostate biopsy or prostatectomy specimen is assigned a first grade, and the second most predominant pattern is assigned a second grade. The Gleason score is determined by adding these two Gleason grades. A Gleason score of 6 is low grade, 7 is intermediate grade, and 8-10 is high grade.

**Figure 8:**
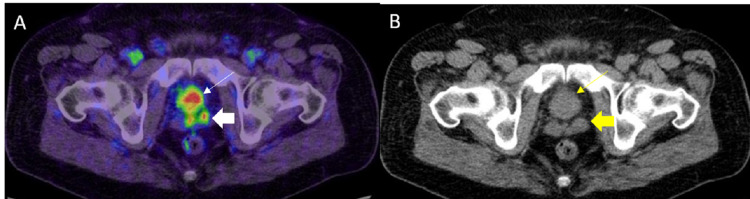
Seminal vesicle invasion A: 68Ga PSMA PET/CT axial image shows uptake in the primary prostate cancer (white thin arrow). Note the invasion of the seminal vesicle (white thick arrow). B: Correlate low-dose axial CT shows the prominent prostate (yellow thin arrow) and seminal vesicle (yellow thick arrow). It is difficult to assess the malignant involvement of the seminal vesicle on this nondiagnostic CT.

**Figure 9:**
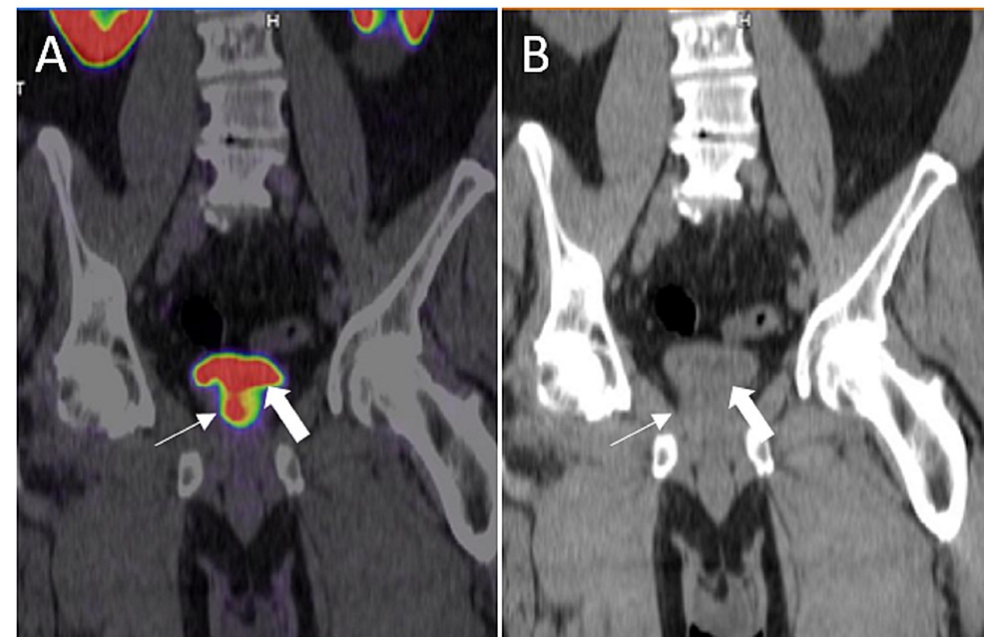
Urinary bladder invasion A: Coronal 68Ga PSMA PET/CT shows primary prostate cancer (white thin arrow) invaginating to the urinary bladder (white thick arrow). B: Correlate coronal CT shows the prominent prostate (white thin arrow) and absent fat plane between the prostate and the urinary bladder (white thick arrow).

**Figure 10:**
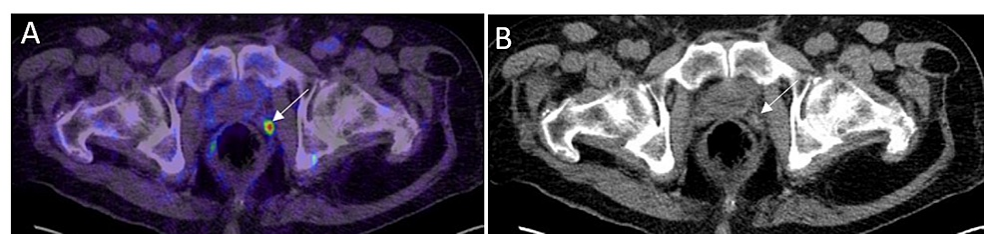
Regional lymph node metastasis A: Axial 68Ga PSMA PET/CT shows metastatic left periprostatic lymph node (white arrow). B: Axial CT shows the correlate small periprostatic lymph node (white arrow).

**Figure 11:**
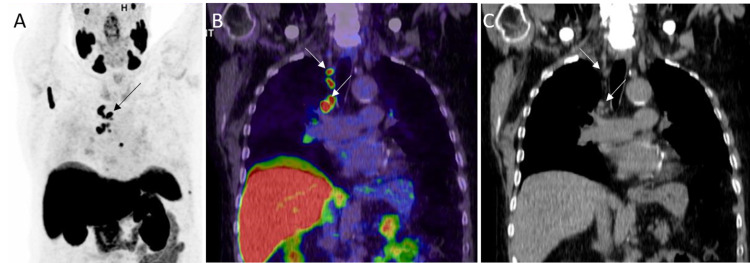
Metastases in mediastinal lymph nodes A: 68Ga PSMA PET MIP image shows intense uptake in the metastatic mediastinal lymph nodes (black arrow). B: Coronal PSMA PET/CT image through the chest and upper abdomen of the same patient shows intense radiotracer uptake in the mediastinal lymph nodes (white arrows). C: Coronal CT image shows the correlate mediastinal lymph nodes (white arrows) that are metastatic.

**Figure 12:**
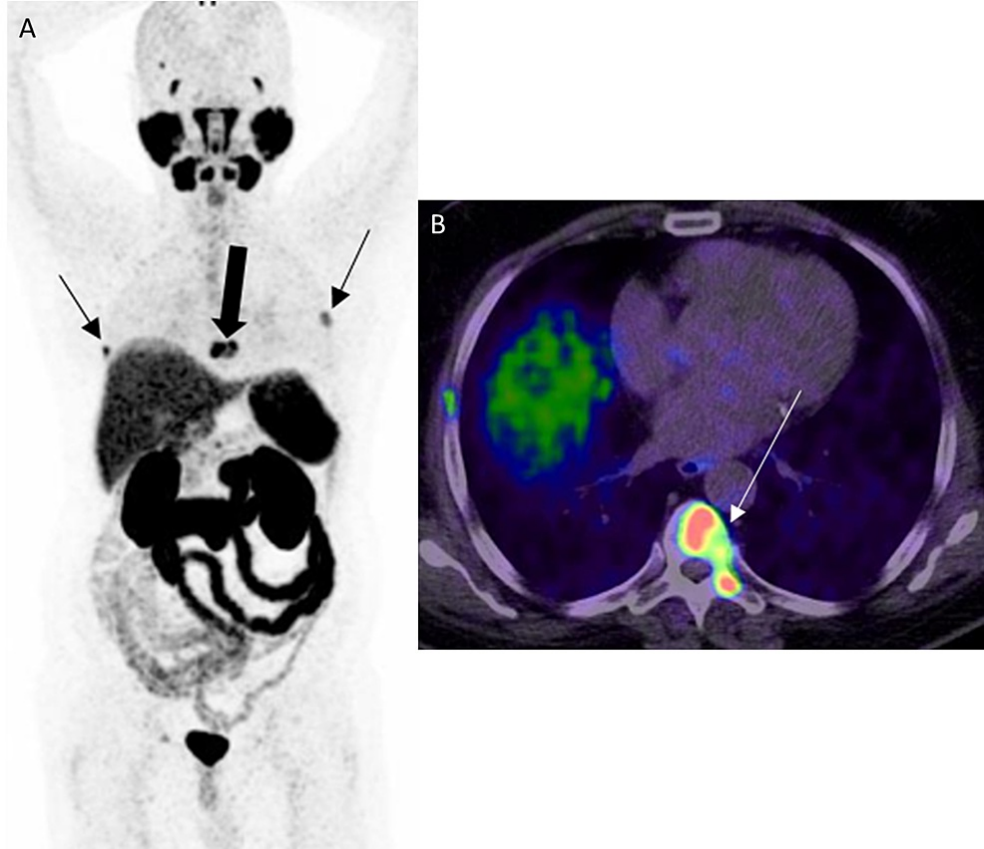
Bone metastases A: 68Ga PSMA PET MIP image shows metastatic bone lesions with focal uptake in bilateral ribs (black thin arrows) and a thoracic vertebra (black thick arrow). B: PET/CT axial image shows intense uptake in the metastatic thoracic vertebra lesion (white arrow) in the same patient.

**Figure 13:**
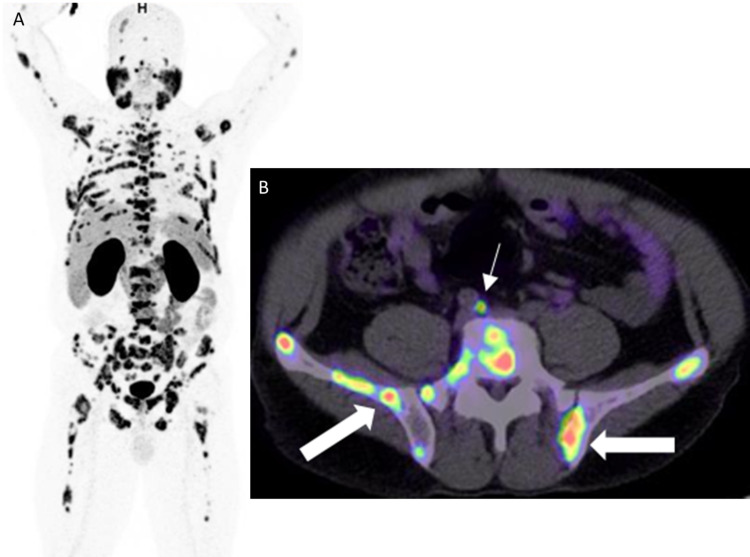
Diffuse bone and iliac lymph node metastases A: 68Ga PSMA PET MIP image shows diffuse bone and iliac lymph node metastases. B: Axial PET/CT image through the pelvis in the same patient shows tracer uptake in the right common iliac lymph node metastasis (white thin arrow) and multiple bone metastases (white thick arrows).

**Figure 14:**
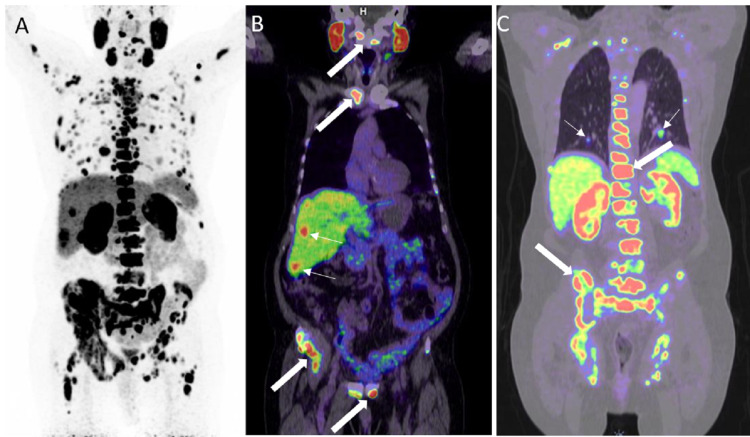
Extensive bone, liver, and lung metastases A: 68Ga PSMA PET MIP image shows extensive metastases in the bones, liver, and lungs. B: Coronal PET/CT in the soft tissue window in the same patient shows liver metastases (white thin arrows). Also, note the multiple bone metastases (white thick arrows). C: Coronal PET/CT in the lung window in the same patient shows bilateral lung metastases (white thin arrows). Also, note the extensive bone metastases (white thick arrows).

The T categories for PCa include clinical T (cT) based on physical examination, prostate biopsy, and imaging and the pathologic T (pT) based on pathology after prostatectomy. pT is likely to be more accurate than cT. The descriptions of T, N, and M are given in Table 3. The same table gives the explanation of the figures given below (Figures 8-14).

**Table 3:** TNM staging of prostate cancer

cT	
T1	Clinically inapparent tumor not palpable or visible by imaging
T1a	Incidental histologic finding in ≤5% of tissue resected at the time of the transurethral resection of the prostate (TURP)
T1b	Incidental histologic finding in >5% of tissue resected at the time of TURP
T1c	Tumor identified by needle biopsy (because of elevated PSA level)
T2	Tumor confined within the prostate
T2a	Tumor involving one-half of one lobe or less
T2b	Tumor involving more than one-half of one lobe, but not both lobes
T2c	Tumor involving both lobes
T3	Tumor extending through the prostatic capsule
T3a	Extracapsular extension (unilateral or bilateral)
T3b	Tumor invading the seminal vesicle(s) (Figure 8A, 8B)
T4	Tumor fixed or invading adjacent structures other than seminal vesicles (Figure 9A, 9B)
pT	
pT2	Organ confined
pT3	Extraprostatic extension
pT3a	Extraprostatic extension or microscopic invasion of the bladder neck
pT3b	Seminal vesicle invasion
pT4	Tumor fixed or invading adjacent structures other than seminal vesicles such as the bladder and rectum
N	
N0	No regional lymph node metastasis
N1	Metastasis in regional lymph node(s) (Figure 10A, 10B)
M	
M0	No distant metastasis
M1	Distant metastasis
M1a	Nonregional lymph nodes such as common iliac lymph nodes and above (Figure 11A-11C)
M1b	Bone(s) (Figure 12A, 12B and Figure 13A, 13B)
M1c	Other site(s) with or without bone disease (Figure 14A-14C)

Other malignant conditions that can show PSMA uptake

**Figure 15:**
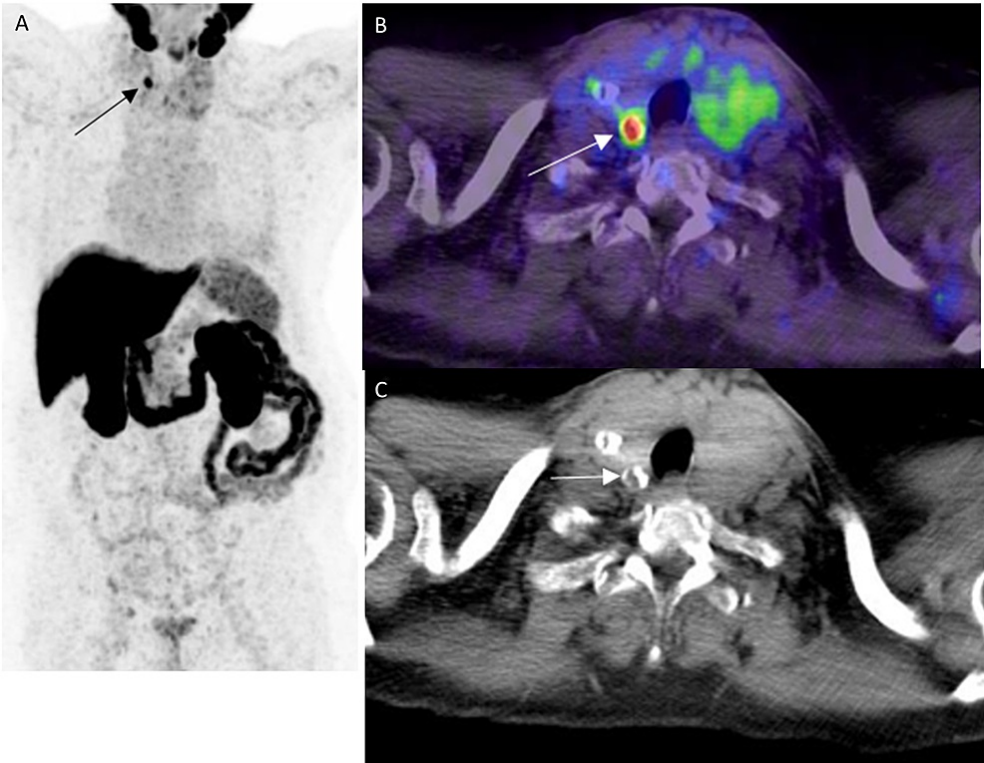
PSMA uptake in thyroid carcinoma A: 68Ga PSMA PET MIP image shows focal uptake in the right lobe of the thyroid (black arrow). B: Axial PSMA PET/CT image through the neck in the same patient shows focal intense uptake in a calcified right thyroid nodule (white arrow). FNA done showed malignant cells. C: Axial CT image through the neck in the same patient shows correlate calcified right thyroid nodule (white arrow).

Many non-prostatic neoplasms express PSMA either on their cell membrane or in the endothelial cells of the capillary beds of tumor neovasculature and consequently show PSMA uptake. Non-prostate malignancies that can show PSMA uptake include renal cell carcinoma, pulmonary adenocarcinoma, multiple myeloma, glioblastoma multiforme, hepatocellular carcinoma, urothelial carcinoma, lymphoma, squamous cell carcinomas, colorectal carcinoma, thyroid carcinoma (Figure 15A-15C), gastrointestinal stromal tumor, and pancreatic neuroendocrine tumor [26].

Benign conditions that can show PSMA uptake

**Figure 16:**
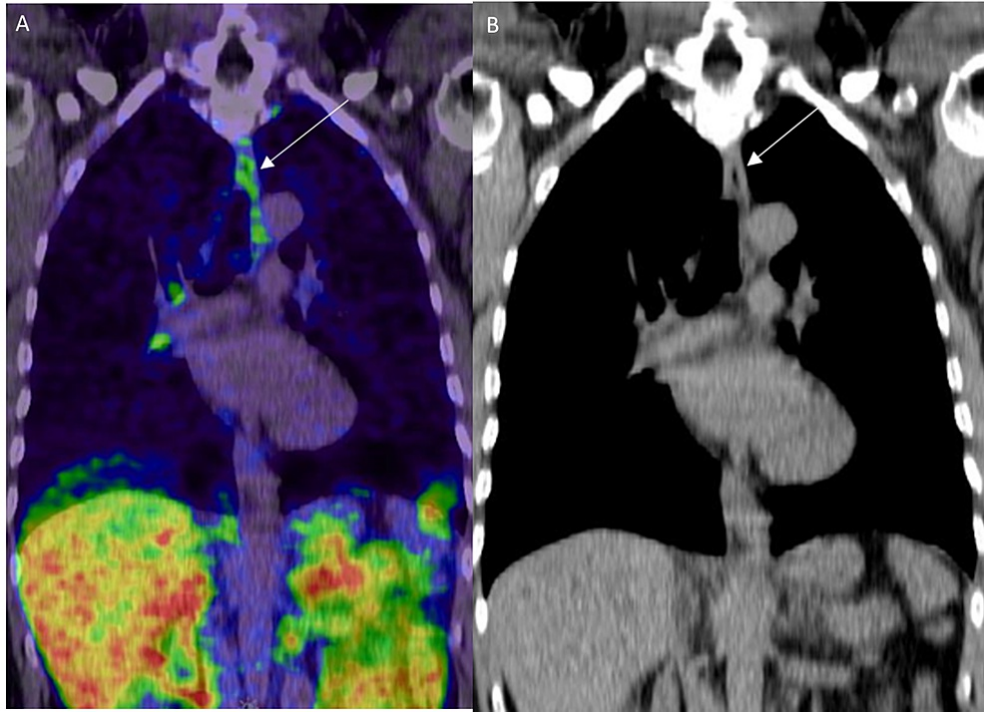
Benign esophageal PSMA uptake A: 68Ga PSMA PET/CT in coronal projection shows mild linear tracer uptake along the esophagus (white arrow) due to inflammation. B: Coronal CT image of the chest in the same patient shows the esophagus (white arrow) with no abnormal wall thickening.

**Figure 17:**
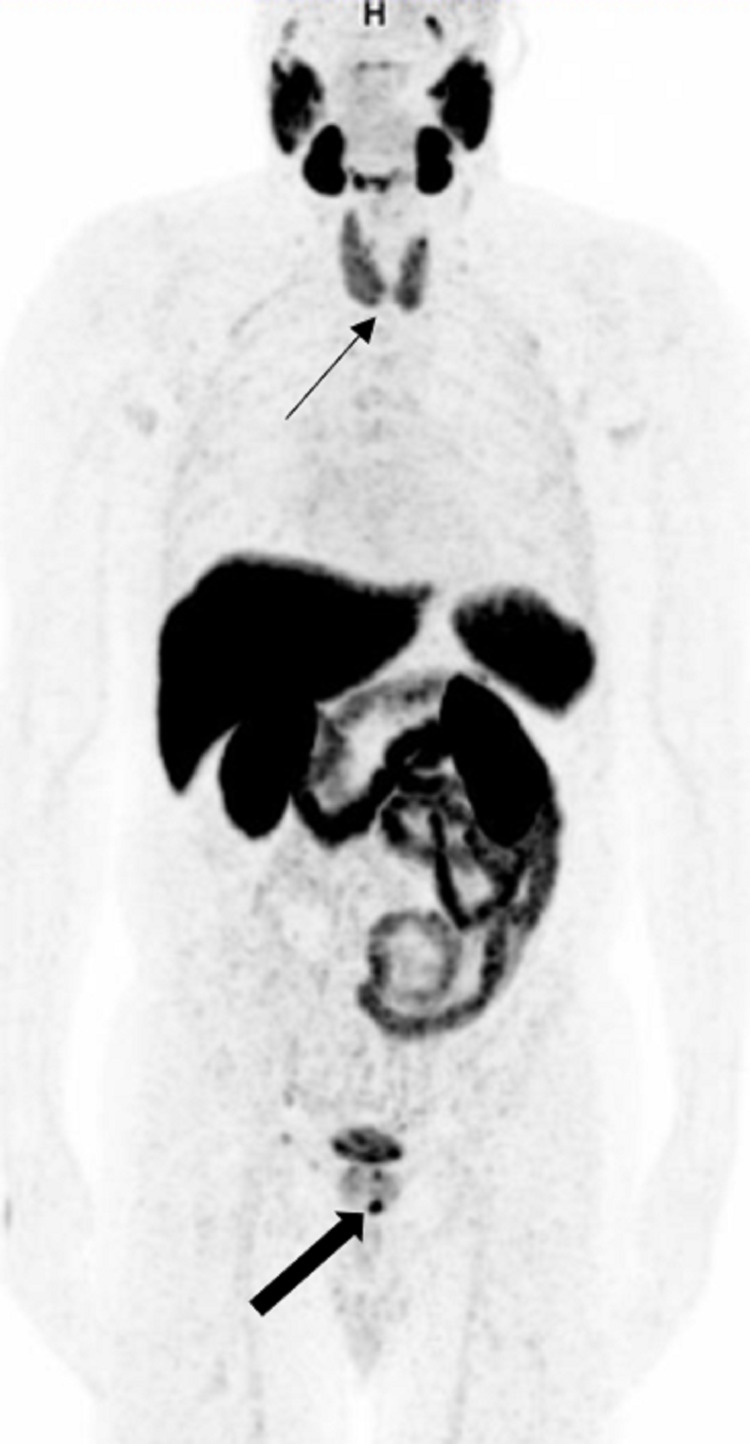
Thyroiditis 68Ga PSMA PET MIP image shows diffuse moderate uptake throughout the bilateral lobes of the thyroid (black thin arrow) likely due to thyroiditis. Note the heterogeneous moderate to high PSMA uptake in the prostate gland involving both lobes with the highest focal uptake on the left (black thick arrow) in this patient with newly diagnosed prostate cancer with PSA level 12.

**Figure 18:**
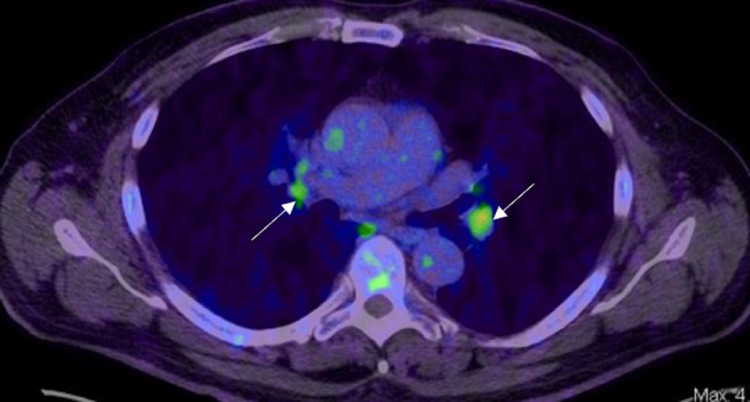
PSMA uptake in granulomatous lymph nodes 68Ga PSMA PET/CT in axial projection through the chest shows mild uptake in the small granulomatous hilar lymph nodes (white arrows).

The following benign conditions can show PSMA uptake: inflammation (Figure 16A, 16B and Figure 17), granulomatous lesions (Figure 18), osteoblastic skeletal conditions (Paget’s disease, fibrous dysplasia, fractures, and degenerative process), hemangioma, gynecomastia, neurogenic tumors such as meningioma, schwannoma, and peripheral nerve sheath tumor, lipid-rich adrenal adenoma, and benign thyroid nodules [26].

Required preliminary information before performing PSMA PET/CT

A summary of the patient’s clinical and oncological history should be provided by the requesting physician. The PET/CT reading physician should explore the indication for the study, PSA and Gleason score in case of primary PCa, PSA level and PSA kinetics in case of BCR, prior definitive treatments such as prostatectomy or radiation therapy, PCa-specific medications such as ADT or other androgen receptor (AR) targeted treatments, and any history of systemic chemotherapy, Radium-223 or Lutetium-177 PSMA RLT. Also, the chart should be reviewed for associated symptoms such as pain, urinary symptoms, or erectile dysfunction. Pertinent comorbidities and any other malignancies should be examined and also prior imaging reviewed [10].

Reporting

The report should describe pertinent clinical data and technical details of the study in a standardized way [27]. Clinical history should include the diagnosis, brief treatment history, reason for the study, and any particular question to be answered. Technical details should include the radiopharmaceutical, injected activity, route, site and time of administration, time of furosemide injection if administered, tracer uptake time period, and body parts that were covered. It should be stated whether the CT component was low dose or diagnostic CT. If a diagnostic CT was performed, a more detailed description of the CT protocol should be provided. Dosimetric parameters can be incorporated as per regulations [10].

While reporting PET findings, attention should be given with regard to the prostate gland or prostate bed, periprostatic structures, regional and distant lymph nodes, bones, liver, and lungs. Regions relating to definite symptoms provided on referral forms should be specifically mentioned. As there is a high chance for small recurrences near the urinary bladder and kidneys to be missed, it is important to evaluate PET images in axial and coronal and sagittal planes and to change the SUV threshold to judge the small abnormal PSMA uptakes. Clear-cut criteria for visual assessment of lesions are not yet established, and PSMA uptake should be reported as low, moderate, or intense in comparison to the background. Semiquantitative measures can be used as per institutional protocol.

The lesions on CT corresponding to the abnormal foci of tracer uptake on PET should be reported. Due to high background activity in the liver, potential liver metastases could be masked on PET. Also, in advanced disease, especially in liver metastases, PSMA expression can be lost likely due to dedifferentiation. Hence, in advanced disease, the diagnostic CT is the mainstay for detecting especially liver metastases, and findings should be detailed in the report. All pathological findings on CT should be described, even if they are PET-negative. Quality issues such as motion artifacts, halo artifacts, and attenuation artifacts should be reported. Comparison with previous imaging should be done and reported if relevant.

The conclusion should address the findings with respect to the clinical questions asked. For PCa imaging, it is suggested to structure the summary to the local tumor involvement in the prostate bed, lymph node metastases, bone metastases, and other probable lesions. If possible, a TNM stage should be provided. Alternatively, an estimate of the probability of diagnosis can be given. If appropriate, differential diagnoses should be discussed. If needed, additional examinations or repeat imaging should be recommended for further lesion clarification [27].

PSMA-RADS version 1.0

Since PSMA can be taken by a variety of benign lesions and non-prostatic malignancies and since the intensity of uptake can differ depending on the level of PSMA expression and location and the size of lesions, a standardized method of interpretation of the scan can aid to reflect the level of certainty on findings [28]. Also, a standardized reporting and data system help the referring physicians to relate the findings efficiently. Furthermore, the systematic categorization of findings allows easier harmonization of data in cases of multicenter studies [29]. Hence, a structured reporting system termed PSMA-RADS version 1.0 was proposed by Rowe et al. [29].

PSMA-RADS-1 (Benign)

PSMA-RADS-1A: This designation describes benign lesions characterized by biopsy or pathognomonic findings on anatomic imaging and without abnormal uptake.

**Figure 19:**
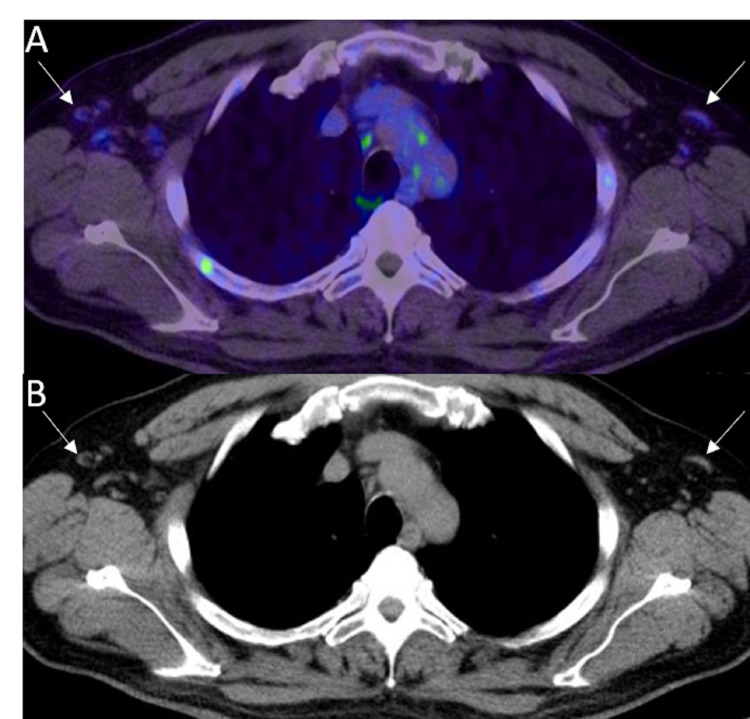
PSMA-RADS-1B A: Axial 68Ga PSMA PET/CT through the chest shows mild uptake in benign small axillary lymph nodes with fatty hila (white arrows). B: Axial CT through the chest in the same patient shows correlate benign small axillary lymph nodes with fatty hila (white arrows).

PSMA-RADS-1B: This includes benign lesions characterized by biopsy or pathognomonic findings on anatomic imaging and with focal radiotracer uptake (Figure 19A, 19B).

PSMA-RADS-2

**Figure 20:**
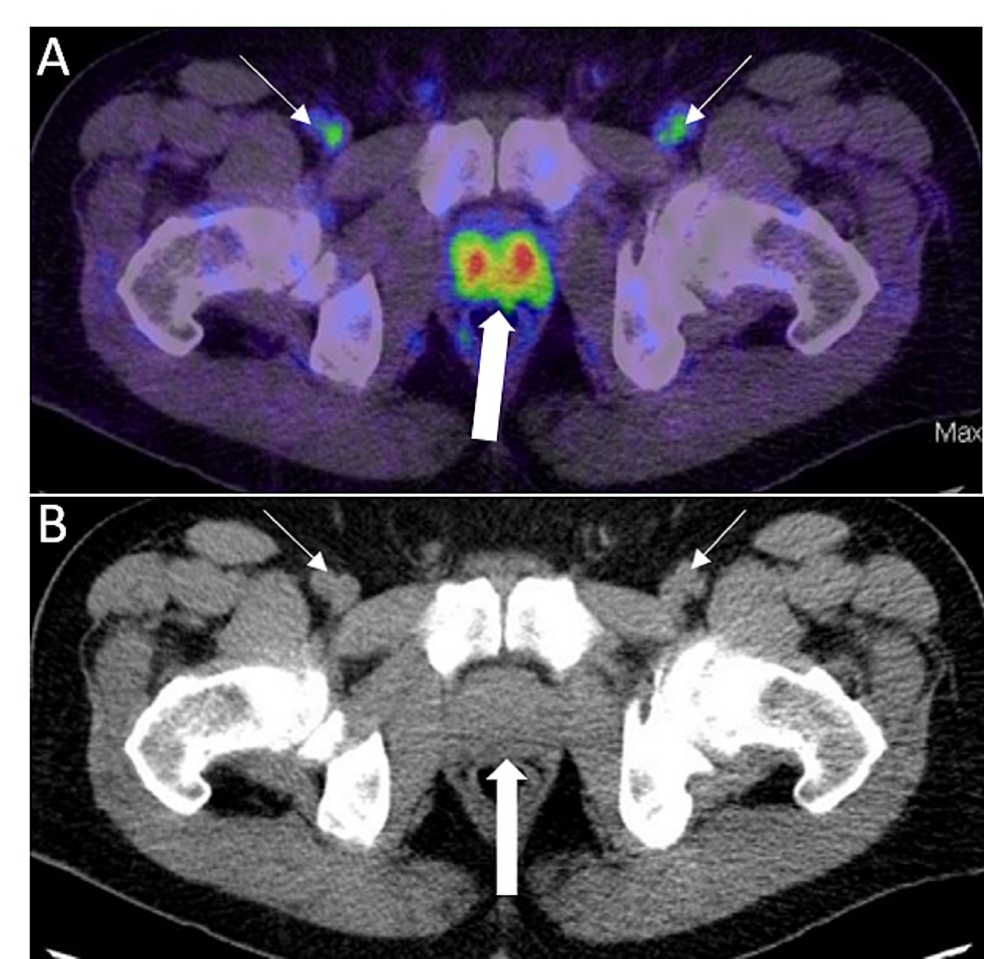
PSMA-RADS-2 A: Axial 68Ga PSMA PET/CT image through the pelvis shows subcentimeter bilateral inguinal lymph nodes with mild uptake (white thin arrows). There was no other pelvic lymphadenopathy in this patient. Note the intense radiotracer uptake in the primary prostate cancer (white thick arrow). B: Axial CT image through the pelvis in the same patient shows correlate subcentimeter bilateral inguinal lymph nodes (white thin arrows). Note the enlarged prostate (white thick arrow).

This describes lesions that are likely benign with equivocal uptake in soft tissues atypical of PCa involvement (e.g., axillary lymph nodes, osseous degenerative change, and small inguinal lymph nodes in the absence of other pelvic lymph node metastasis) (Figure 20A, 20B) and in bone lesion atypical of PCa involvement.

PSMA-RADS-3

These are lesions with equivocal uptake requiring further workup.

PSMA-RADS-3A: These are lesions with equivocal uptake in soft tissues typical of PCa involvement, such as pelvic or retroperitoneal lymph nodes. Biopsy or follow-up imaging can help confirm the diagnosis.

**Figure 21:**
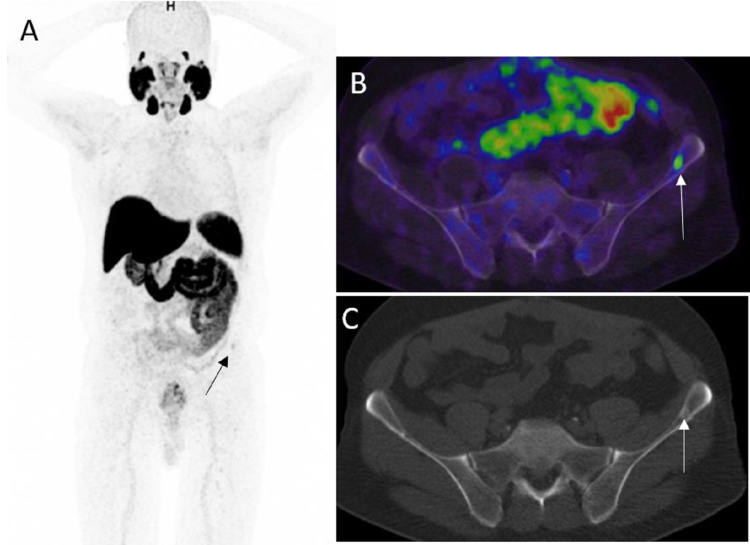
PSMA-RADS-3B A: 68Ga PSMA PET MIP image shows a single focus of abnormal uptake in the left ilium (black arrow). B: 68Ga PSMA PET/CT axial image through the pelvis shows small focal radiotracer uptake in the left ilium (white arrow). C: CT axial image through the pelvis in the same patient shows the correlate small indeterminate sclerotic lesion in the left ilium (white arrow).

PSMA-RADS-3B: This include lesions with equivocal uptake in bone lesion that are not clearly benign (Figure 21A-21C). In comparison to a bone scan, MR, biopsy, or follow-up imaging may confirm the diagnosis.

**Figure 22:**
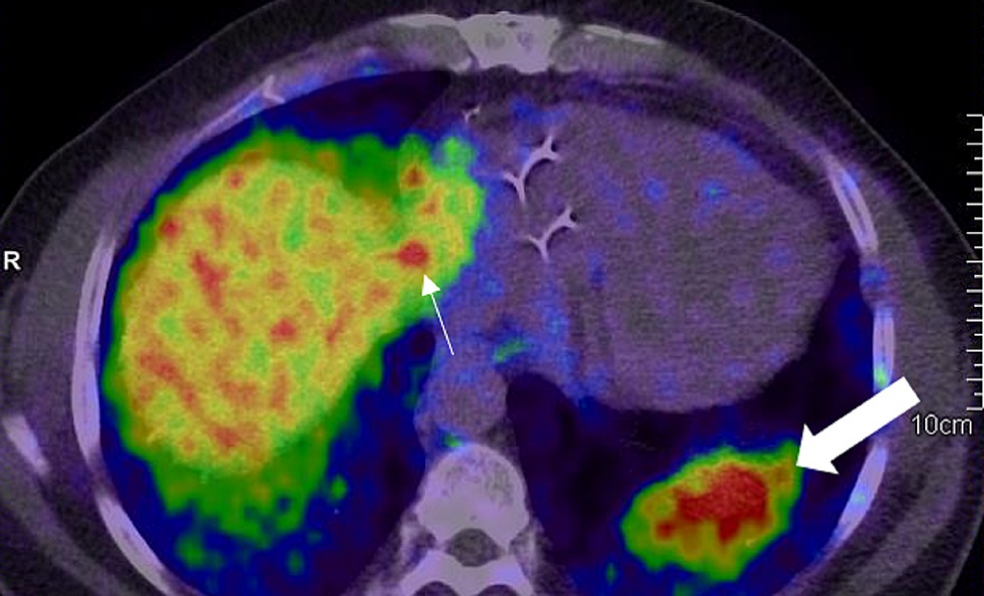
PSMA-RADS-3C Axial 68Ga PSMA PET/CT image of the upper abdomen shows a focal uptake in the left lobe of the liver (white thin arrow) that is indeterminate. There was no obvious correlate lesion on the low-dose CT. Further follow-up is required for clarification. Note the physiological uptake in the spleen (white thick arrow).

PSMA-RADS-3C: This designation includes lesions with intense uptake in site, highly atypical of PCa, with a likelihood of non-prostate malignancy (Figure 22) or benign tumor. Biopsy or organ-specific follow-up imaging can help confirm the diagnosis.

PSMA-RADS-3D: These are lesions suggestive of malignancy on anatomic imaging but lacking uptake (e.g., non-prostate malignancy, neuroendocrine PCa, and uncommon cases of PCa that do not express PSMA). Biopsy or organ-specific imaging can be done.

PSMA-RADS-4

PCa is highly likely. This describes lesions with intense uptake in site, typical of PCa, but lack definitive findings on conventional imaging (e.g., intense uptake in a retroperitoneal node, which is <1 cm in short axis on CT).

PSMA-RADS-5

PCa is almost certainly present. These are lesions with intense uptake in site, typical of PCa, with corresponding findings on conventional imaging.

In cases where there is only a limited number of lesions, each lesion can be given a PSMA-RADS designation. In cases of widespread disease, demarcation of every lesion is unfeasible and not necessary. In such cases, PSMA-RAD designation for a dominant or representative lesion, such as a lesion with the highest uptake or largest size, can be provided. Whatever the number of lesions, providing an overall PSMA-RADS designation for the scan can help the referring physician obtain a general impression that reflect the likelihood of the presence of PCa. Usually, the highest PSMA-RADS lesion will also be the overall PET scan designation [28,29].

Patient radiation exposure from 68Ga PSMA PET/CT

The total radiation exposure will be a combination of the exposure from PET radiopharmaceutical and the CT study. The average coefficient for the effective dose of 68Ga PSMA is 2.0 × 10−2 mSv/ MBq, with a resultant average effective dose of 3 mSv for a 150 MBq administered activity [10]. Studies have shown that the kidneys, bladder wall, and salivary and lacrimal glands receive the most absorbed dose [30]. The dose from the CT scan depends on the CT systems and protocol used. The effective CT dose ranges from 1 to 20 mSv depending on the CT protocol.

## Conclusions

68Ga PSMA PET/CT is a noninvasive imaging technique for PCa. It is indicated in cases of BCR in patients already treated with radical prostatectomy or radiotherapy, to primary stage patients with a high risk of primary PCa before definitive therapy, in monitoring treatment response, and to target biopsies. PSMA targeting PET demonstrates much higher sensitivity than conventional imaging in detecting residual, recurrent, or metastatic disease, thus leading to key changes in patient management. PSMA is expressed in a variety of normal tissues and benign and other malignant tumors. Understanding the normal and variants of biodistribution of PSMA is crucial to avoid misinterpretations.
